# Oxygen‐regulated post‐translation modifications as master signalling pathway in cells

**DOI:** 10.15252/embr.202357849

**Published:** 2023-10-25

**Authors:** Michael Batie, Temitope Fasanya, Niall S Kenneth, Sonia Rocha

**Affiliations:** ^1^ Department of Biochemistry, Cell and Systems Biology, Institute of Molecular Systems and Integrative Biology University of Liverpool Liverpool UK

**Keywords:** 2‐oxoglutarate dioxygenases, hydroxylation, hypoxia, methylations, PTMs, Metabolism, Post-translational Modifications & Proteolysis, Signal Transduction

## Abstract

Oxygen is essential for viability in mammalian organisms. However, cells are often exposed to changes in oxygen availability, due to either increased demand or reduced oxygen supply, herein called hypoxia. To be able to survive and/or adapt to hypoxia, cells activate a variety of signalling cascades resulting in changes to chromatin, gene expression, metabolism and viability. Cellular signalling is often mediated via post‐translational modifications (PTMs), and this is no different in response to hypoxia. Many enzymes require oxygen for their activity and oxygen can directly influence several PTMS. Here, we review the direct impact of changes in oxygen availability on PTMs such as proline, asparagine, histidine and lysine hydroxylation, lysine and arginine methylation and cysteine dioxygenation, with a focus on mammalian systems. In addition, indirect hypoxia‐dependent effects on phosphorylation, ubiquitination and sumoylation will also be discussed. Direct and indirect oxygen‐regulated changes to PTMs are coordinated to achieve the cell's ultimate response to hypoxia. However, specific oxygen sensitivity and the functional relevance of some of the identified PTMs still require significant research.

## Graphical abstract

Cellular signalling is often mediated via post‐translational modifications, also in response to hypoxia. This review discusses the impact of changes in oxygen availability to PTMs and how such oxygen‐regulated changes are coordinated to achieve cellular responses to hypoxia.

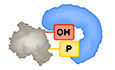

## Introduction

Oxygen is critical for multicellular organisms' viability as it is required for efficient energy production by cells in mitochondrial respiration via the oxidative phosphorylation pathway (Taylor & Pouyssegur, 2007). Given its importance, cells have evolved complex and exquisite sensing and response mechanisms to reduced oxygen availability, hereafter named hypoxia. Hypoxia can occur via problems in oxygen supply (such as vascular obstruction) and/or via excessive demand by metabolically active cells (such as active muscle contraction in exercise; Kenneth & Rocha, 2008).

In cells, hypoxia activates a potent signalling cascade, culminating in the stabilisation and activation of the transcription factor family hypoxia inducible factor (HIF) (Smith *et al*, 2008). HIF (an obligatory heterodimer of HIF‐α and HIF‐1β) then transactivate a variety of genes spanning several important pathways, including oxygen restoration, glycolysis, cell proliferation, cell growth, cell death, transcription regulation as well as negative regulators (Reviewed in Missiaen *et al*, 2023). In mammalian cells, there are three HIF‐α genes: HIF1A (HIF‐1α), EPAS1 (HIF‐2α), and HIF3A (HIF‐3α). HIF‐1α is ubiquitously expressed across all cells investigated, but HIF‐2α and HIF‐3α have more restricted expression in certain tissues. They all have non‐redundant functions; however, HIF‐1α and HIF‐2α do share several genes (Loboda *et al*, 2010).

Changes to oxygen tensions are transmitted to HIF via the action of specific enzymes called prolyl hydroxylases, belonging to a larger family of 2‐oxoglutarate and iron‐dependent dioxygenases (2‐OGDs) (Reviewed in Wilson *et al*, 2020). Hydroxylation of HIF‐α subunits in their oxygen‐dependent degradation domain generates a high affinity binding site for recognition by the tumour suppressor Von Hippel–Lindau Tumour Suppressor (VHL), which is part of an E3‐ubiquitin ligase, thus promoting ubiquitination and degradation by the proteasome (Ivan *et al*, 2001; Jaakkola *et al*, 2001; Yu *et al*, 2001). Another 2‐OGD also controls HIF directly, but in this case by reducing HIF transcriptional potential. This is mediated by factor inhibiting HIF‐1 (FIH, gene name *HIF1AN*) that promotes hydroxylation of an asparagine residue in the C‐terminus transactivation domain of HIF‐α. FIH‐mediated hydroxylation of HIF‐α subunits results in inhibition of binding by the co‐activator proteins p300/creb‐binding protein (CBP; Peet & Linke, 2006), reducing HIF transactivation potential for certain genes (Kasper *et al*, 2005). Genetic studies in mice have determined that as much as 40% of HIF‐dependent genes are also regulated by FIH (Kasper *et al*, 2005).

Structural work on FIH hydroxylase domain identified a novel structural fold (Elkins *et al*, 2003; Trewick *et al*, 2005), which then led to the discovery of other enzymes that contained similar structural properties resulting in the classification of the 2‐OGD family. 2‐OGD members thus require 2‐oxoglutarate, iron and oxygen for their activity (Wilson *et al*, 2020). Amongst this family, many different activities are present including hydroxylases, lysine‐demethylases and arginine demethylases. As such, all these post‐translation modifications have the potential to be altered directly by changes in the oxygen level in the cell.

## Direct oxygen‐dependent PTMs

**Figure 1:**
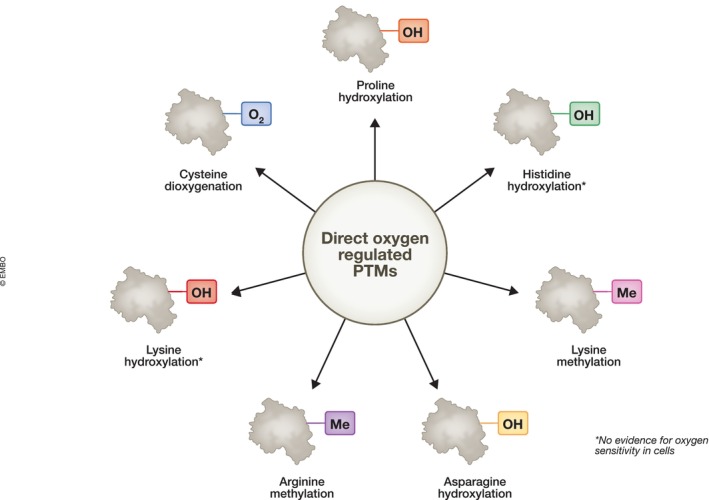
Protein modifications with confirmed and potential direct oxygen‐dependent regulation Oxygen, acting as a cofactor for 2‐OGDs, can directly regulate proline hydroxylation, asparagine hydroxylation, lysine methylation, arginine methylation and cysteine deoxygenation. In addition, it has the potential to directly alter lysine hydroxylation and histidine hydroxylation; however, oxygen sensitivity has not been investigated.

Given that oxygen is a co‐factor for a variety of enzymes, many PTMs are directly modulated by oxygen levels, these will be reviewed below, with a focus on cellular oxygen sensing and the response to hypoxia in mammalian systems (Fig 1).

### Proline hydroxylation

As mentioned above, HIF‐α hydroxylation of key prolines in the oxygen‐dependent degradation domain of HIF‐α subunits creates a high affinity binding site for VHL (Ivan *et al*, 2001; Jaakkola *et al*, 2001; Yu *et al*, 2001). There are three prolyl hydroxylase enzymes that have been shown to target HIF‐α in humans: prolyl hydroxylase domain‐containing protein 1 (PHD1) (gene name *EGLN2*), PHD2 (gene name *EGLN1*) and PHD3 (gene name *EGLN3*) (Fandrey *et al*, 2006). Oxygen affinities have been measured *in vitro*, revealing significant sensitivity of these enzymes to reductions in oxygen availability, making these optimal cellular oxygen sensors (Wilson *et al*, 2020). In cells, PHD2 is the dominant HIF‐1α hydroxylase (Berra *et al*, 2003). This was also confirmed in genetics studies in the mouse, with PHD2 deletion resulting in embryological lethality (Takeda *et al*, 2008). Additional studies revealed that PHDs have different binding preferences for specific HIF‐α isoforms (Appelhoff *et al*, 2004). PHD1 shows preference for HIF‐2α in some cells, while PHD3 equally targets HIF‐1α and HIF‐2α (Appelhoff *et al*, 2004).

### Proline hydroxylation outside of HIFs

**Figure 2:**
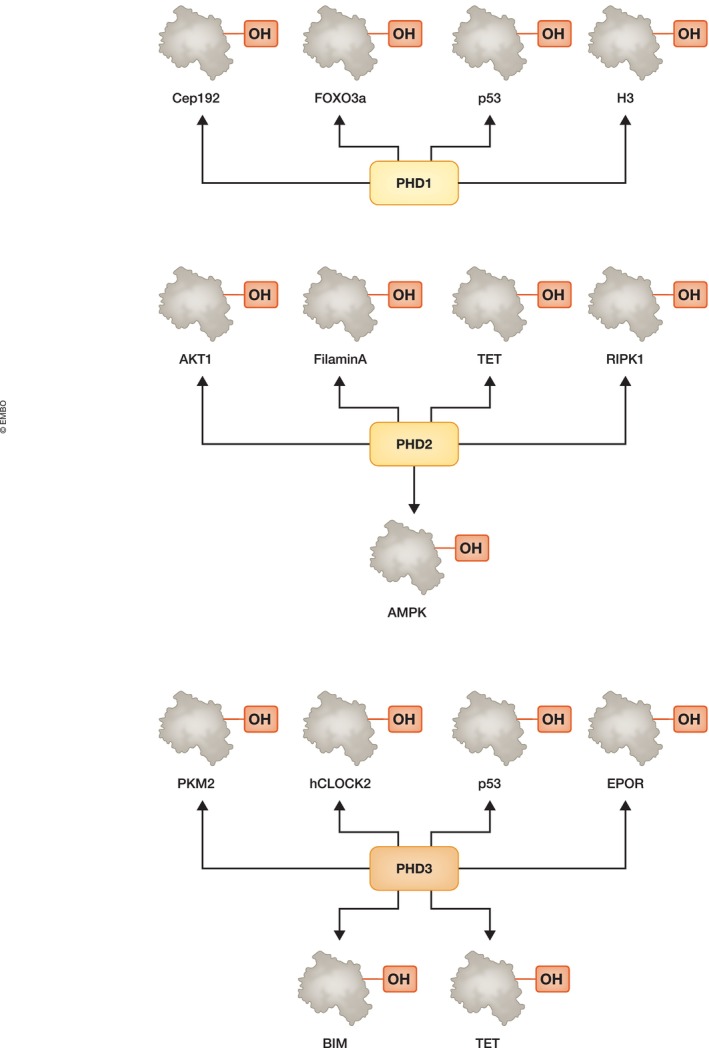
PHD targets identified outside HIF transcription factors PHD1 controls targets associated with transcription and cell cycle. PHD2 controls pathways associated with growth, metabolism and proliferation, while PHD3 targets are varied such as transcription factors and chromatin regulators, DNA damage response and cell proliferation and death.

Proline hydroxylation is very abundant in collagen and important for its synthesis (Salo & Myllyharju, 2021); however, progress in identification of additional targets for PHDs outside HIF‐α subunits has been surrounded by controversy (Bersten & Peet, 2019; Cockman *et al*, 2019; Strowitzki *et al*, 2019). Technical difficulties due to mass spectrometry misassignments due to a lack of robust assays and reagents have resulted in problems determining the identity and/or veracity of the modified residues. Furthermore, the lack of a known proline dehydroxylase has led to the hypothesis that this modification should only occur in short lived proteins. However, modelling data on the HIF signalling pathway suggests that such activity does exist in cells (Nguyen *et al*, 2013). Despite these difficulties, several studies using state of the art mass spectrometry analysis, combined with specific addition of PHD inhibitors and genetic analysis in cells have identified several new targets of all three PHD enzymes (Fig 2) and more targets are being identified (Data ref: Jiang *et al*, 2023a). Work developed by the Von Kriegsheim group resulted in a method to trap PHDs and FIH interacting partners, all of which are potential targets (Rodriguez *et al*, 2016). Interestingly, different PHDs seem to have roles in distinct cellular processes. PHD1 targets have broad association with cell cycle control and transcriptional regulation, including Forkhead Box O3 (FOXO3a) (Zheng *et al*, 2014), centrosomal protein 192 (Cep192) (Moser *et al*, 2013), p53 (Ullah *et al*, 2017) and histone H3 (Liu *et al*, 2022). PHD2 targets are associated with growth, metabolism and proliferation signalling pathways including targets such as AKT serine/threonine kinase 1 (AKT1; Guo *et al*, 2016), filamin A (Segura *et al*, 2016) and ten eleven translocation (TET) (Fan *et al*, 2020). PHD2 was shown to hydroxylate receptor interacting protein kinase 1 (RIPK1), which is required for its degradation in normoxia (Zhang *et al*, 2023). RIPK1 is important for the processes of inflammation and necroptosis (Zhang *et al*, 2023). More recently, AMP‐activated protein kinase (AMPK) was shown to be hydroxylated by PHD2 in mitochondria, a hydroxylation that is required for AMPK release from this organelle and results in VHL‐dependent degradation (Jiang *et al*, 2023b). In the absence of PHD2 activity, PHD2 and AMPK remain bound in the mitochondria resulting in AMPK activation and metabolic modulation of cancer cells (Jiang *et al*, 2023b). PHD3 targets seem to be the most varied including mitogen‐activated protein kinase 6 (MAPK6; Rodriguez *et al*, 2016); p53 (Rodriguez *et al*, 2018), hClock2 (Xie *et al*, 2012), pyruvate kinase M1/2 (PKM2; Luo *et al*, 2011), erythropoietin receptor (EPOR; Heir *et al*, 2016), Bim (Li *et al*, 2019) and TET (Fan *et al*, 2020).

Adding to the controversy surrounding new targets of PHDs, in mice, only PHD2 is essential for development, while PHD1 and PHD3 are viable but have discrete phenotypes (Takeda *et al*, 2006; Aragones *et al*, 2008). Of course, mice and humans differ significantly in several aspects, but one aspect to consider is genetic mouse models are usually kept in very well‐controlled conditions, away from microorganisms, with access to food and water and their metabolic rate is very different from humans (Seok *et al*, 2013). It is thus possible that individual PHDs are required for stress responses such as response to inflammation or even for neuronal function. These are aspects that have not been explored in any depth in the PHD1 and PHD3 knockout mice. Of note, it has been shown that PHD1 deletion protects mice from inflammatory bowel disease (Tambuwala *et al*, 2010). Furthermore, mutations in PHD1 have been detected in rare cases of human cancer such as pheochromocytoma (Yang *et al*, 2015). As such, as methods develop and data is available on proteome wide analysis of proline hydroxylation, more biology will be found as a direct consequence of oxygen‐dependent proline hydroxylation (Box 1). Furthermore, for PHD targets that are present in high copy number, proline hydroxylation‐specific antibodies can be produced as exemplified in the case of H3‐P16 hydroxylation (Liu *et al*, 2022) and AMPK‐P188 hydroxylation (Jiang *et al*, 2023b) antibodies.

Box 1 **In need of answers**

- Is there a proline de‐hydroxylase in cells?
- Do additional PHD targets outside the HIF system exist and are these targets tissue specific?
- Is histidine and lysine hydroxylation sensitive to oxygen in cells and what are their biological functions?
- What are other KDM targets sensitive to oxygen outside histones?
- Does hypoxia induce global changes to ubiquitination in cells?
- Which dioxygenase controls SENP activity in hypoxia?

### Asparagine hydroxylation

Factor inhibiting HIF (FIH) promotes hydroxylation of asparagine residues in the c‐terminal transactivation domain of HIF‐1α and HIF‐2α (Lando *et al*, 2002). Unlike proline hydroxylation, targets of FIH outside the HIF factors have been readily identified by mass spectrometry approaches (Cockman *et al*, 2009a; Rodriguez *et al*, 2016). In fact, in this case, even evidence for a de‐hydroxylase activity existing in cells has been provided, even though its identity has remained elusive (Rodriguez *et al*, 2020). FIH has been shown to mediate the hydroxylation of asparagine residues in many different proteins, including ankyrin‐containing proteins such as nuclear factor kappa B subunit 1 (NFKB1), NFKB2, NFKB inhibitor alpha (NFKBIA; Cockman *et al*, 2009b; Singleton *et al*, 2011) and deubiquitinases such as OTU deubiquitinase, ubiquitin aldehyde binding 1 (OTUB1; Scholz *et al*, 2016) and Cezanne (Mader *et al*, 2020). Although for some of these targets, the biological function of the asparagine hydroxylation is unknown, for others hydroxylation creates a binding site for other proteins, changing the functional context of the acceptor protein (Scholz *et al*, 2016; Mader *et al*, 2020).

It is well known that FIH's affinity for molecular oxygen is very high, indicating that FIH will only be inhibited in severe conditions of hypoxia (Wilson *et al*, 2020). Interestingly, FIH deletion in mice does not lead to embryological lethality, but rather results in metabolic changes (Zhang *et al*, 2010). This suggests that FIH, like PHD1 and PHD3, is not required for development but rather is required for other important processes once the organism is born.

### Histidine hydroxylation

FIH has also been shown to hydroxylate histidine residues in ankyrin repeat contain proteins such as tankyrase‐2 (Yang *et al*, 2011); however, the functional significance of this modification remains unknown. Since then, other members of the 2‐OGD family have been shown to have histidinyl hydroxylase activity (Bundred *et al*, 2018). These include nucleolar protein 66 (NO66) and MYC‐induced nuclear antigen (MINA; Ge *et al*, 2012). The functional significance of these enzymes is clear, with important roles in regulating ribosome biology and hence protein translation (Bundred *et al*, 2018). However, whether they are oxygen sensitive in cells has yet to be determined fully, so additional research into these areas is needed (Box 1).

### Lysine hydroxylation

Much like proline hydroxylation, lysine hydroxylation has been seen extensively in collagen (Salo & Myllyharju, 2021). Jumonji‐C (JmjC) domain containing proteins are also part of the 2‐OGD family (Shmakova *et al*, 2014). Jumonji domain containing 4 (JMJD4) was shown to hydroxylate eukaryotic release factor 1 (eRF1; Feng *et al*, 2014). Functionally, this modification is important for efficient translation termination (Feng *et al*, 2014). JMJD6 was originally associated with apoptosis but since then has had many associated biological functions, including transcription and RNA splicing (Kwok *et al*, 2017). JMJD6 has reported lysine hydroxylase and arginine demethylase activities (Kwok *et al*, 2017), although this has been debated (Bottger *et al*, 2015; Islam *et al*, 2019). Recent work by the Ratcliffe group identified several proteins that become hydroxylated via the action of JMJD6 (Cockman *et al*, 2022). Most proteins identified as lysine hydroxylation, possessed this modification in lysine‐rich regions, predicted to be unstructured (Cockman *et al*, 2022), suggesting once again that hydroxylation can change structure and function, possibly by creating new binding sites or by preventing alternative PTMs on the lysine residues (Wang & Cole, 2020). However, this needs further validation, and use of structural work. Previously, JMJD6 was also shown to hydroxylate lysines on p53, which results in p53 inhibition (Wang *et al*, 2014). Finally, JMJD7 has been shown to hydroxylate lysines in developmentally regulated GTP‐binding protein 1 (DRG1) and DRG2 (Markolovic *et al*, 2018). However, the functional significance of this modification remains unknown. Of note, oxygen sensitivity of these 2‐OGD enzymes still requires investigation both *in vitro* and in cells, so it is not known if lysine hydroxylation is regulated by hypoxia.

### Lysine methylation

In addition to specific PTMs requiring oxygen for their addition to proteins, some can require oxygen for their removal. Lysine methylation on histones is one of the most well‐characterised histone modifications and is involved in fine‐tuning gene expression (Kouzarides, 2007). Some histone lysine methylations are associated with gene transitional activation, such as H3K4me3 and H3K36me3; others are associated with gene transcriptional repression, such as H3K9me3 and H3K27me3. As with most PTMs, histone methylation is a dynamic event, being promoted by histone methyl‐transferases, and removed by histone demethylases (Hyun *et al*, 2017). Lysine demethylation is catalysed by JmjC domain‐containing proteins and lysine‐specific demethylases. The latter group only displays histone lysine demethylation activity for H3K9me1/me2 and H3K4me1/me2, whereas JmjC domain‐containing proteins can demethylate a more diverse range of histone lysine methylations (Kooistra & Helin, 2012). There are 32 identified JmjC domain‐containing proteins in humans, which are divided based on target selectivity and sequence homology into subfamilies (lysine specific demethylase (KDM)2‐KDM8), and 23 of them display demethylase activity in cells (Shmakova *et al*, 2014).

**Figure 3:**
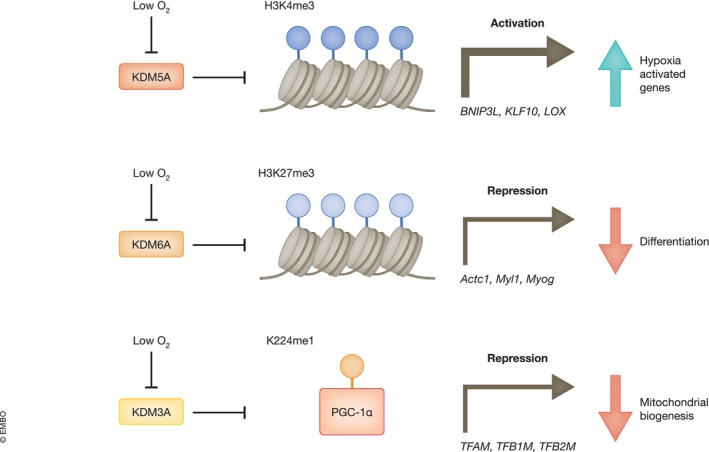
Lysine methylation and oxygen sensing Oxygen‐dependent inactivation of KDM6A in hypoxia is linked to H3K27me3 hypermethylation and gene repression of differentiation genes and reduced differentiation. Oxygen‐dependent inactivation of KDM5A in hypoxia is linked to H3K4me3 hypermethylation and gene activation of hypoxia‐activated genes. Oxygen‐dependent inactivation of KDM3A in hypoxia is linked to increased PGC‐1α K224me1 and gene repression of mitochondrial biogenesis genes and reduced mitochondrial biogenesis.

It is well established, using immunoblotting, immunofluorescence, and mass spectrometry techniques, that hypoxia triggers an increase in the total levels of several histone modifications (Kindrick & Mole, 2020; Wilson *et al*, 2020; Frost *et al*, 2021). Furthermore, loci‐specific changes in histone lysine methylation for H3K4me3, H3K27me3, H3K9me3 and H3K36me3 on a genome‐wide scale in response to hypoxia have been characterised by several groups using ChIP‐sequencing, and these changes correlate with changes in gene expression in response to hypoxia (Adriaens *et al*, 2016; Prickaerts *et al*, 2016; Batie *et al*, 2019; Chakraborty *et al*, 2019; Ortmann *et al*, 2021). JmjC domain‐containing proteins are 2‐OGDs, and whilst there is a range of mechanisms at play with regards to the dynamic crosstalk between histone lysine methylation and hypoxia (Choudhry & Harris, 2018; Kindrick & Mole, 2020; Ortmann *et al*, 2021; Liu *et al*, 2022). There is evidence for direct cellular oxygen sensing by JmjC domain‐containing proteins contributing to hypoxia‐induced changes in the histone methylation landscape (Fig 3). Inhibition of the H3K27 demethylase KDM6A in hypoxia drives gene repression through increased H3K27me3 at a group of hypoxia‐repressed gene promoters (Chakraborty *et al*, 2019). KDM6A was shown to be oxygen sensitive over physiologically relevant oxygen concentrations in cells and *in vitro*; two residues in the active site of KDM6A (M1190 and E1335) confer its high oxygen sensitivity, and these sites are not conserved in the other H3K27 demethylase, KDM6B (Chakraborty *et al*, 2019). Furthermore, hypoxia‐induced increases in H3K4me3 and gene expression at a subset of hypoxia upregulated genes has been attributed to inhibition of KDM5A in hypoxia (Zhou *et al*, 2010; Batie *et al*, 2019), molecular mechanisms conferring KDM5A oxygen sensitivity over other KDM5 proteins are still under investigation. Interestingly, a recent study has described an interplay between PHD1‐mediated hydroxylation and KDM5A targeting to genomic loci (Liu *et al*, 2022). Here, PHD1 hydroxylated histone H3 at proline 16, which was necessary for KMD5A binding to these modified histones. However, how PHD1 targets histone H3 at specific loci is still unknown.

There is also evidence for cellular oxygen sensing linked to increased H3K9me3 in hypoxia by the H3K9 demethylase KDM4A (Dobrynin *et al*, 2017; Hancock *et al*, 2017), and *in vitro* oxygen affinity assays highlight the potential for other JmjC domain‐containing proteins, such as KDM4B (Chakraborty *et al*, 2019), to sense oxygen in physiological settings. In addition to histone lysine demethylation, other lysine demethylation targets are emerging for JmjC domain‐containing proteins. For example, KDM2A has been shown to directly demethylate the NF‐κB subunit p65, controlling its activity in cancer cells (Lu *et al*, 2010) and β‐catenin, regulating its stability in the nucleus (Lu *et al*, 2015). Furthermore, Qian *et al* (2019) found that KDM3A demethylates monomethylated K224 on PPARG coactivator 1 alpha (PGC‐1α), and that oxygen sensing by KDM3A regulates PGC‐1α‐mediated mitochondrial biogenesis via this modification. This study demonstrates that direct oxygen‐sensitive lysine methylation, coordinated by JmjC domain‐containing proteins, expands beyond histones and represents an exciting new area of study (Box 1).

### Arginine methylation

Proteins are also methylated on arginine residues, and this PTM is implicated in a wide range of biological processes (Wu *et al*, 2021). Arginine methylation is less well characterised than histone methylation, particularly pertaining to demethylation. In a seminal study, *in vitro* biochemical analyses identified a subset of JmjC domain‐containing proteins as possessing arginine demethylase catalytic activity against histone and non‐histone substrates (Walport *et al*, 2016), in addition to their lysine demethylase activity. One of these, KDM5C, has recently been reported to demethylate the autophagy regulator Unc‐51 like autophagy activating kinase 1 (ULK1) at R170 in an oxygen sensitive manner, with hypoxia impairing KDM5C arginine demethylase activity, increasing ULK1 R170me2s, and promoting hypoxia‐induced autophagy (Li *et al*, 2022). KDM3B has also been reported as a histone arginine demethylase targeting H4R3me2s (Li *et al*, 2018), although the role of hypoxia/oxygen sensing in regulating this modification was not explored. The added complexity of dual arginine/lysine demethylase activities of JmjC domain‐containing proteins provides a challenge in delineating the plethora of molecular mechanisms and biological functions related to these enzymes, particularly in the context of oxygen sensing.

### N‐terminal cysteine oxidation

Oxygen‐sensitive PTMs are not exclusively regulated by 2‐OGDs. In the cysteine branch of the N‐degron pathway, oxidation of N terminal cysteines promotes subsequent arginylation and protein degradation (Varshavsky, 2019). N‐terminal cysteine oxidation catalysed by plant cysteine dioxygenases represents an oxygen‐sensing mechanism in plants, which is important for their hypoxic adaptation (Gibbs *et al*, 2011; Licausi *et al*, 2011; Weits *et al*, 2014; White *et al*, 2017). Masson *et al* (2019) identified 2‐aminoethanethiol dioxygenase (ADO), a thiol dioxygenase, as a direct cellular oxygen sensor, which catalyses the dioxgenation of N‐terminal cysteines in humans. In this study, ADO was shown to target several proteins, including regulator of G protein signalling 4 (RGS4), RGS5 and Interleukin 32 (IL32), which are stabilised in hypoxia via ADO inhibition. ADO‐mediated N‐degron regulation likely extends to other targets.

### Indirect oxygen‐regulated PTMs

**Figure 4:**
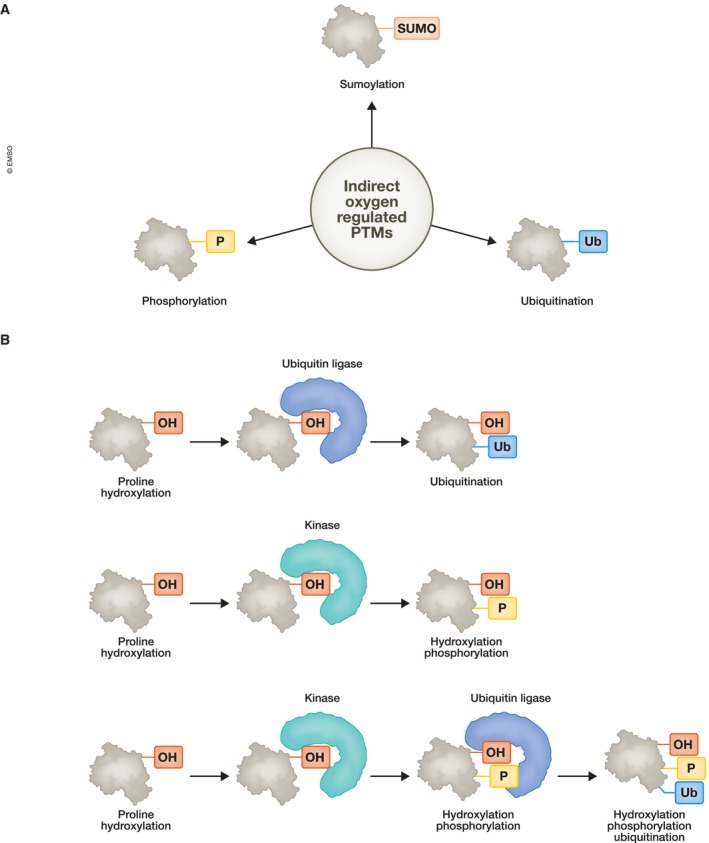
Protein modifications indirectly controlled by oxygen (A) Changes to oxygen have been shown to control phosphorylation, ubiquitination and sumoylation via indirect processes, some of which required 2‐OGDs control over kinases, deubiquitinases and SUMO proteases. (B) Crosstalk of Proline hydroxylation with phosphorylation and ubiquitination. Proline hydroxylation has been shown to recruit kinases and ubiquitin ligases resulting in dual modification and even triple modifications of proteins.

Although not directly controlled by oxygen, other PTMs play important roles in the cellular response to oxygen reductions. Here phosphorylation, ubiquitination and sumoylation will be discussed (Fig 4A). Other PTMs such as acetylation are also important, but genome wide research is limited (Collier *et al*, 2023) and as such will not be described here.

### Phosphorylation

Protein phosphorylation is crucial to regulate many of the signalling pathways induced in normal and cancerous hypoxic cells (Batie *et al*, 2022). One of the best‐described roles of kinase‐dependent signalling pathways in the hypoxic response is the energy‐sensing pathways that induce an adaptive response to maintain cellular homeostasis (Lee *et al*, 2020a). These include AMPK, mechanistic target of rapamycin (mTOR) and the activation of the unfolded protein response (UPR): all key kinase‐dependent, energy‐sensing signalling pathways that are responsive to hypoxic stress (Wouters & Koritzinsky, 2008; Ivanova *et al*, 2019; Dengler, 2020). AMPK is activated by two main mechanisms during hypoxia: by liver‐kinase B1 (LKB1) when AMP/ATP ratio rises due to reduced oxidative phosphorylation in mitochondria and by calcium/calmodulin‐dependent protein kinase kinase 2 (CaMKK2) as a consequence of calcium release from the ER (Hardie, 2007; Dengler, 2020). CaMK2 activation during hypoxia also leads to changes in the gene expression profile of cells independently of HIF through activation of NF‐κB signalling (Culver *et al*, 2010).

AMPK activation during hypoxia results in reprogramming of the cellular energy consumption rates in part by inhibiting another protein kinase, the mammalian target of rapamycin complex 1 (mTORC1; Liu *et al*, 2006; Wouters & Koritzinsky, 2008; Dengler, 2020; Chun & Kim, 2021). mTORC1 is a protein kinase complex, which is a key signalling node for the detection of nutrients and energy availability during many cellular stresses, including oxygen deprivation (Saxton & Sabatini, 2017). AMPK‐dependent mTORC1 inhibition decreases protein synthesis within the cell by inhibiting the downstream target S6 kinase (S6K) and other key translation initiation factors during hypoxic stress (Liu *et al*, 2006; Wouters & Koritzinsky, 2008; Saxton & Sabatini, 2017; Chun & Kim, 2021). In addition to the AMPK and mTOR axis, the UPR is a parallel kinase‐dependent signalling pathway through which hypoxic stress can limit cellular energy consumption (Wouters & Koritzinsky, 2008; Bartoszewska & Collawn, 2020). The UPR is a conserved signalling pathway that maintains cellular homeostasis in response to the accumulation of unfolded or misfolded proteins in the endoplasmic reticulum (ER; Jain, 2017; Read & Schroder, 2021). One of the key arms of the UPR is mediated by PRKR‐like endoplasmic reticulum kinase (PERK), a transmembrane receptor kinase resident in the ER (Jain, 2017; Read & Schroder, 2021). PERK is activated by severe hypoxia and phosphorylates eukaryotic translation initiation factor 2A (eIF2α) to suppress *de novo* protein synthesis to protect cells until oxygen levels are restored (Koumenis *et al*, 2002; Wouters & Koritzinsky, 2008). However, hyper‐activation of PERK in hypoxic cells by pharmacological ER‐stress inducers leads to suppression of HIF activity by blocking translation of HIF1α and sensitises cells to hypoxic stress (Ivanova *et al*, 2018). The co‐ordinated response of these key kinase‐dependent signalling pathways leads to global changes in cellular energy homeostasis by suppressing anabolic processes (such as protein synthesis) and activating catabolic processes to restore the cellular ATP supply (Wouters & Koritzinsky, 2008; Chun & Kim, 2021).

Although targeted approaches have led to fundamental discoveries of kinase‐dependent signalling pathways associated with energy metabolism, the understanding of the role of phosphorylation‐dependent signalling during hypoxia is still superficial. Indeed, a recent review characterising the post‐translational modifications of just the HIF subunits reveal many layers of regulation that are not yet understood (Reviewed in Albanese *et al*, 2020). In addition to HIF‐dependent pathways, several studies have employed unbiased whole proteome phosphoproteomics to study global changes to protein phosphorylation in response to hypoxic stress. Phosphoproteomic analyses of hypoxia‐treated pulmonary artery smooth muscle cells revealed large‐scale changes to the phosphoproteome identifying 331 significantly changed phosphoproteins, from a total of 2,347 identified phosphoproteins, representing a change of phosphorylation of approximately 14% of all identified phosphosites (Luo *et al*, 2022). If this data is extrapolated to consider the 72,000 high confidence Ser/Thr/Tyr phosphosites identified in the human proteome (Kalyuzhnyy *et al*, 2022), there may be as many as 10,000 hypoxia‐induced changes to the phosphoproteome. The limited hypoxia‐dependent phosphoproteomic experiments performed to date have revealed changes in phosphorylation of proteins in pathways as diverse as mitogen‐activated protein kinase pathways (ERK1 and ERK2), regulation of TGF (tumour growth factor)‐β receptor signalling, changes to Rho GTPase signalling and key factors involved in cytoskeletal organisation (Nilsson *et al*, 2010; Datta *et al*, 2021; Luo *et al*, 2022). These suggest that multiple kinase/phosphorylation‐dependent signalling pathways exist in tandem to modulate the cellular response in low oxygen, to tailor the response to different cellular contexts.

The knowledge regarding the effects of hypoxia on phosphatase activity is also limited. Protein phosphatase 1 (PP1) has been shown to be involved in hypoxic preconditioning in rat hearts (Ladilov *et al*, 2002). Furthermore, PP1 nuclear targeting subunits (PNUTS) is hypoxia inducible (Lee *et al*, 2007), being responsible for reduced PP1 activity over MDM2 proto‐oncogene (MDM2) (Lee *et al*, 2007). Protein phosphatase 2 phosphatase activator (PP2A) activity in hypoxia is modulated by HIF‐dependent and independent mechanisms (Elgenaidi & Spiers, 2019), while protein phosphatase 4 catalytic subunit (PP44C) has been shown to be increased in all tissues of rats exposed to high altitude (Ma *et al*, 2023). HIF‐1α is also a possible target for dephosphorylation by Protein Phosphatase, Mg^2+^/Mn^2+^ Dependent 1G (PPM1G), leading to its destabilisation (Pyo *et al*, 2018) but other phosphatases are also potentially involved in the control of HIF levels and activity, directly and indirectly (Wang *et al*, 1995). From multiple transcriptomic studies performed in cells exposed to hypoxia, it is clear that hypoxia can induce the levels of dual specificity phosphatases (DUSPs): DUSP1 (Tiana *et al*, 2018; Ortmann *et al*, 2021), DUSP5 (Ortmann *et al*, 2021), DUSP6 (Tiana *et al*, 2018); DUSP9 (Frost *et al*, 2019; Ortmann *et al*, 2021) and DUSP10 (Ortmann *et al*, 2021). Finally, hypoxia promotes the degradation of DUSP7 (Li *et al*, 2014). However, the biological roles of DUSPs in hypoxia are not well investigated.

### Crosstalk of phosphorylation with hydroxylation

Recent studies have provided evidence for a crosstalk between proline hydroxylation and phosphorylation (Fig 4B). Lee *et al* (2020b) demonstrated that PHD1‐mediated hydroxylation primed CMGC kinases for autophosphorylation and activation, exemplified by dual specificity tyrosine‐(Y)‐phosphorylation regulated kinase 1 (DYRK1) kinases. Crosstalk between proline hydroxylation and phosphorylation is also suggested in the hydroxylation of AKT1 by PHD2 that promotes VHL binding without degradation of AKT1, but with reduced phosphorylation (Guo *et al*, 2016). PHD2 was also recently shown to hydroxylate AMPK, allowing for its release from mitochondria and degradation via VHL, thus reducing AMPK levels and activity in normoxia (Jiang *et al*, 2023b). Although not formally addressed, the hydroxylation of Cep192 by PHD1 resulted in increased binding by S‐phase kinase associated protein 2 (SKP2; Moser *et al*, 2013). SKP2 normally requires phosphorylation events for binding (Cai *et al*, 2020), so it is possible that proline hydroxylation is required for phosphorylation event(s) at Cep192 prior to SKP2 binding; however, this has not been demonstrated. These studies suggest an interesting crosstalk between oxygen‐dependent and independent PTMs.

### Ubiquitination

Ubiquitination is a versatile post‐translational modification, which regulates diverse fundamental features of protein substrates. The covalent conjugation of ubiquitin to proteins, either as a monomer or in ubiquitin chains, regulates protein stability, localisation and interaction with other molecules within the cell (Swatek & Komander, 2016). Ubiquitin is mediated by a three‐step enzymatic cascade, with an E1 ubiquitin‐activating enzyme, E2 ubiquitin‐conjugating enzymes and E3 ubiquitin, while it is removed by de‐ubiquitinase enzymes (DUBs; Neutzner & Neutzner, 2012). For a comprehensive review on DUBs in hypoxia, please see the article by Mennerich *et al* (2019).

Ubiquitin‐mediated proteasome degradation is key to the central mechanism required for the adaptive response to hypoxia mediated by HIF (Choudhry & Harris, 2018; Lee *et al*, 2020a). The conjugation of lysine 48‐linked ubiquitin chains to HIFα subunits by an E3 ubiquitin ligase complex containing the von Hippel–Lindau (VHL) tumour suppressor protein is the primary mechanism of control of the stability of HIFα subunits (Maxwell *et al*, 1999). In addition to VHL the E3 ubiquitin ligases, MDM2, F‐Box and WD repeat domain containing 7 (FBXW7), BRCA1 DNA repair associated (BRCA1) and spliceosome associated factor 1, recruiter of U4/U6.U5 Tri‐SnRNP (SART1) have all been reported to conjugate degradative ubiquitin chains to HIFα subunits to suppress the hypoxic response (Kang *et al*, 2006; Koh *et al*, 2008; Cassavaugh *et al*, 2011; Joshi *et al*, 2014). Although degradative ubiquitination is important for HIF regulation, the E3 ubiquitin ligases TNF receptor associated factor 6 (TRAF6) and X‐linked inhibitor of apoptosis (XIAP) can conjugate non‐canonical lysine 63‐linked ubiquitin chains to HIF‐1α to increase HIF activity by controlling stability and subcellular localisation, respectively (Sun *et al*, 2013; Park *et al*, 2017). In addition, lysine 11 chains are required for full HIF levels and activity (Bremm *et al*, 2014; Moniz *et al*, 2015).

HIF activity can also be indirectly regulated by protein ubiquitination with the ubiquitin ligases Siah1/2, which are able to target both PHD1 and PHD3 for proteasomal‐mediated degradation in hypoxic cells (Nakayama *et al*, 2004). Ubiquitination is clearly essential for the HIF‐mediated responses to hypoxia, but it also has additional HIF‐independent roles in the hypoxic response. Pathway analysis of hypoxia‐dependent signalling pathways in smooth muscle cells reveals changes in phosphoproteins in the ubiquitin proteasome pathway, indicating major global changes to the hypoxia‐dependent proteome (Luo *et al*, 2022). In addition, global mRNA levels are altered in hypoxia due to hypoxia‐dependent linear ubiquitination of Argonaute RISC Catalytic Component 2 (AGO2) by the linear ubiquitin chain assembly (LUBAC) complex. Ubiquitinated AGO2 restrains miRNA‐mediated gene silencing to have global effects on the global accumulation of mRNAs, likely having great impact on many cell signalling pathways (Zhang *et al*, 2021). The impact of hypoxia‐mediated ubiquitination on the hypoxic response is likely to affect multiple aspects of cell signalling, with the true extent of its control only revealed by multi‐omics‐based experiments (reviewed in (Luo *et al*, 2022)), which are still emerging.

### Crosstalk of ubiquitination with hydroxylation

An interesting link between ubiquitination and hydroxylation also exists (Fig 4B). In addition, several DUBs are hydroxylated by FIH, including OTUB1 (Scholz *et al*, 2016) and Cezanne, a Lysine 11 specific DUB (Mader *et al*, 2020). This suggests that indirectly, oxygen can control ubiquitination levels of different lysine chains to substrates in the cell. Additional hydroxylation in proteins such as Cep192 (Moser *et al*, 2013) or FOXO3a (Zheng *et al*, 2014) interfere with binding of either an E3‐ligase (SKP2) or a DUB (Ubiquitin Specific Peptidase 9 X Linked [USP9X]), respectively. Similarly, hydroxylation of MAPK6 by PHD3 potentially leads to its dissociation from HECT, UBA, and WWE domain containing E3 ubiquitin protein ligase 1 (HUWE1) and results in increased stability of MAPK6 (Rodriguez *et al*, 2016). Interestingly, hydroxylation of AKT1 by PHD2 results in VHL binding and suppresses AKT phosphorylation and activation, without altering protein levels (Guo *et al*, 2016). The authors suggested that VHL does not require its ligase activity since depletion of cullin 2 did not recapitulate VHL depletion (Guo *et al*, 2016). However, further research is needed to fully address this point.

No direct effect on ubiquitin ligase or deubiquitinase activity by hypoxia has been reported. Rather, indirect effects mediated by hydroxylation of the enzymes (OTUB1, Cezanne) or their targets (HIF, Cep192, FOXO3a, AKT1 and AMPK) have been demonstrated. Approaches using di‐Gly antibody combined with mass spectrometry (Wagner *et al*, 2011) analysis in cells or tissues exposed to hypoxia would provide a more global or unbiased view of the effects of reduced oxygen in the ubiquitination cycle (Box 1).

### Sumoylation

Sumoylation is another important protein PTM discovered more than two decades ago, which involves the covalent addition of small ubiquitin‐like modifier (SUMO) protein (around 12 kDa) through its C‐terminus to lysine residues of a target substrate (Celen & Sahin, 2020; Wang *et al*, 2021). So far, five SUMO isoforms have been identified in mammals, of which SUMO 1–3 have been well studied for their roles in stress response and cellular physiology (Zhao, 2018), whereas little is known about SUMO 4–5 (Baczyk *et al*, 2017; Li *et al*, 2017). SUMO 2 and 3 differ only in three amino acids (~ 97% similarity) and so are often referred to as SUMO2/3 (Cimarosti *et al*, 2012; Wang *et al*, 2016).

SUMO conjugation modifies proteins that are involved in transcriptional regulation, gene expression, maintenance of genome integrity, chromatin organisation, nuclear transport and DNA damage (Gareau & Lima, 2010; Iribarren *et al*, 2018). Like the ubiquitination process, SUMO conjugation is a multi‐step enzyme‐mediated complex, made up of SUMO‐activating enzyme (SAE1/2 dimer), which activates the matured SUMO protein, SUMO conjugating enzyme (Ubc9) and a ligase (E3). However, unlike ubiquitination, Sumoylation does not necessarily result in substrate degradation, and several reports have supported the role of Sumoylation in enhancing protein stability/prevention of protein degradation via the proteasome (Shao *et al*, 2004; Culver *et al*, 2010). SUMO conjugation to a protein can be reversed by a family of eight enzymes called SUMO/Sentrin‐specific proteases (SENPs) made up of SENP1, SENP2, SENP3, SENP5, SENP6, SENP 7 and SENP8, which catalyse deconjugation and release of SUMO from its substrates (Nayak & Muller, 2014; Kunz *et al*, 2018), whereas SENP8 functions in neural precursor cell expressed developmentally downregulated protein 8 (NEDD8) pathway (Mendoza *et al*, 2003).

Several studies have reported changes to the ‘SUMO proteome’ or sumoylation of proteins under both acute and chronic low oxygen tension (hypoxia) in cells and in the context of a whole organism. Studies in cells exposed to acute hypoxia showed upregulation of SUMO‐1 or SUMO2/3 conjugates, exposure to chronic hypoxia, in addition, showed upregulation of many SUMO targets (Chachami *et al*, 2019). Earlier work by Comerford and colleagues in 2003 demonstrated that hypoxia‐increased SUMO‐1 expression resulted in a global increase in SUMO conjugation (Comerford *et al*, 2003), this was backed up by another study on the brains and hearts of hypoxic mice (Shao *et al*, 2004). In 2004, Shao and colleagues reported an increase in SUMO‐1 and SENP‐1 mRNA levels following exposure of adult male and female mice to 10% oxygen (Shao *et al*, 2004), whereas Cimarosti and colleagues in 2012 demonstrated increased expression of both SUMO‐1 and SUMO‐2/3 following oxygen/glucose deprivation in hippocampal neurons at the protein level but observed no increase in SENP‐1 mRNA (Cimarosti *et al*, 2012). This suggests that differences in cells are important for determining the mechanism behind sumoylation in hypoxia. This study also identified a possible neuroprotective role for SENP1, as overexpression of SENP1 resulted in reduced sumoylation and consequently induced neuronal death, following oxygen/glucose deprivation (Cimarosti *et al*, 2012). In addition, changes to SUMO‐conjugation have also been reported under ischaemic conditions (Loftus *et al*, 2009; Datwyler *et al*, 2011; Yang *et al*, 2012). A recent study used a proteomics‐based approach to identify two groups of sumoylation targets under hypoxic conditions: proteins with increased sumoylation and expression, and those with no change in expression with or without changes in sumoylation status. The former category consists mostly of known HIF‐1α target genes and glycolytic enzymes such as phosphoglycerate kinase 1 (PGK1), enolase 1 (ENO1) and lactate dehydrogenase A (LDHA), which suggests a role for SUMO conjugation in glycolysis, while the latter were mostly transcription factors or transcription regulators (Chachami *et al*, 2019). A strong interplay has been established between sumoylation and hypoxia signalling, as SUMO conjugation has been shown to regulate the activities of main mediators of the hypoxia signalling such as HIF‐1α, HIF‐2α, PHD, FIH, VHL, p300/CPB (Nunez‐O'Mara & Berra, 2013) and regulated HIF‐1β transcription activities *in vitro* (Tojo *et al*, 2002). Regulation of HIF signalling through sumoylation has been comprehensively reviewed by Filippopoulou *et al* (2020) and will not be further discussed here.

Hypoxia has been shown to modulate sumoylation mostly via control of SENP activity (Culver *et al*, 2010; Kunz *et al*, 2016). A study investigating the activation of NF‐κB in hypoxia identified a mechanism by which hypoxia‐induced signalling cascade leading to inhibitor of κBα (IκBα) phosphorylation but did not result in its canonical proteasomal degradation (Culver *et al*, 2010). The mechanism behind this stabilisation was assigned to reduced SENP activity, as NF‐κB activation of certain targets could be mimicked by SENP5‐7 depletion (Culver *et al*, 2010). More recently, work from the Muller laboratory identified that hypoxia specifically reduced SENP3‐5 activity in cells, which can be reversed by 30 min re‐oxygenation (Kunz *et al*, 2016). This raises the possibility of SENPs being targets of specific 2‐OGDs. This possibility would widen the reach of 2‐OGDs into yet another signalling pathway, that of sumoylation. However, at present, there is no information if this does indeed happen, and the nature of the modification is also unclear. Thus, further research is needed in this area.

### Conclusions and future perspectives

While it is clear that oxygen directly controls many different signalling pathways by its role as a cofactor in the action of 2‐OGDs and ADO, much is still left to be uncovered (see Box 1). This includes elucidation of new targets of oxygen‐dependent enzymes/PTMs, a better understanding of the factors determining cellular oxygen sensitivities, and how specificity is achieved regarding the hypoxia response in more widespread PTMs controlled by oxygen‐sensitive enzymes. With the increased sophistication, sensitivity and accessibility of proteomics approaches and the continuing development of chemical, genetic and molecular biology tools to better study PTMs, this represents an area of hypoxia research with great potential for new discoveries. In addition, the quest for the elusive de‐hydroxylase continues and such a finding would complement our knowledge of the dynamics of the hydroxylation mechanisms in hypoxia. A major challenge will be understanding how these PTMs crosstalk in the context of oxygen sensing, both between each other, and with indirect oxygen‐regulated PTMs, including the ones mentioned in this review, phosphorylation, ubiquitination and sumoylation.

## Author contributions

**Michael Batie:** Formal analysis; investigation; writing – original draft; writing – review and editing. **Temitope Fasanya:** Investigation; writing – original draft; writing – review and editing. **Niall S Kenneth:** Formal analysis; investigation; writing – original draft; writing – review and editing. **Sonia Rocha:** Conceptualization; supervision; funding acquisition; writing – original draft; project administration; writing – review and editing.

## Disclosure and competing interests statement

The authors declare that they have no conflict of interest.
